# Health-related quality of life among people living with HIV/AIDS in Togo: individuals and contextual effects

**DOI:** 10.1186/s13104-019-4171-x

**Published:** 2019-03-15

**Authors:** Issifou Yaya, Lihanimpo Djalogue, Akouda Akessiwè Patassi, Dadja Essoya Landoh, Ayélé Assindo, Aboubakari Nambiema, Kanfitine Kolani, P’Niwè Massoubayo Patchali, Essodjèlouna Manani Bignandi, Abdoulahy Diallo, Didier Koumavi Ekouévi, Bayaki Saka

**Affiliations:** 1 Centre Africain de Recherche en Epidémiologie et en Santé Publique (Caresp-Togo), Lomé, Togo; 2 Service de Médecine Interne, Centre Hospitalier Universitaire (CHU) Kara, Kara, Togo; 3 Service de Maladies Infectieuses, CHU Sylvanus Olympio, Université de Lomé, Lomé, Togo; 4 World Health Organization (WHO), Country Office of Togo, Lomé, Togo; 5 Service de Dermatologie et IST, CHU Sylvanus Olympio, Université de Lomé, B.P: 30785, Lomé, Togo; 6 Service de Gynécologie-Obstétrique, Clinique Biasa Lomé, Togo; 7 Division de la Santé Communautaire, Ministère de la Santé, Lomé, Togo; 8 Service de Médecine Générale, Centre Hospitalier Régional (CHR) Tomdè, Kara, Togo

**Keywords:** Quality of life, PLWHA, Togo

## Abstract

**Objective:**

The objective of this study is to assess the quality of life and to identify factors associated with good global quality of life among people living with HIV/AIDS (PLWHA) in Togo.

**Results:**

In total, 880 PLWHA with mean age (standard deviation) of 39.6 (10.1) years, were interviewed. Most of them (78.4%) were female. The global score of quality of life was ranged from 42.6 to 112, with a mean (standard deviation) estimated at 86.3 ± (13.3). More than the three-quarters (76.2%) of the participants had a good global quality of life. In multivariate analysis, secondary education level or higher (adjusted odds ratio = 1.78, 95% confident interval (CI) [1.10–2.85]), living in Kara health region (adjusted odds ratio = 4.39, 95% CI [2.94–6.57]), being on antiretroviral therapy (adjusted odds ratio = 6.99, 95% CI [4.11–11.9]) and HIV sero-status disclosure (adjusted odds ratio = 1.83, 95% CI [1.28–2.61]) were associated with a better overall quality of life (score ≥ 77.3).

## Introduction

In sub-Saharan Africa, a great deal of effort and intervention has been implemented for people infected with HIV, ranging from awareness raising on prevention to the generalization of antiretroviral treatment (ART). In Centrale and Western Africa, about 2.1 million (35%) of PLWHA had access to ART in 2016 [1]. These therapies, by restoring and preserving the immune system, thus contribute to a considerable improvement in the expectations and quality of life (QOL) of PLWHA. To date HIV infection is considered as a chronic disease and measuring health-related quality of life (HRQOL) is increasingly known as a very relevant to assess the follow-up judgment criterion of PLWHA, particularly the effectiveness of the therapeutic strategies.

Several instruments, which were established with validity and reliability, have been used to assess the HRQOL of PLWHA, including the World Health Organization quality of life scale, brief version (WHOQOL-HIV BREF) [2].

In the scientific literature, a large number of studies assessed the quality of PLWHA in different developing countries [3–8]. Most of them have highlighted the positive role of ART in improving the quality of life of PLWHA. For example, in Nigeria, Bello et al. [7] showed that among PLWHA in a secondary health care facility the HRQOL was positively associated with the duration of ART. Other factors such as socio-demographic characteristics and clinical outcomes could influence the HRQOL. In addition, behavioral factors, such as alcohol use, drug consumption, smoking or risky sexual behavior have also been reported to be associated with HRQOL [9, 10].

Although several studies have been conducted particularly among PLWHA in Togo, very little knowledge is available about the QOL in this population. In addition, assessing QOL of PLWHA is useful and important to appreciate the quality of the health care they receive in the health facilities during their follow-up [10]. To overcome this gap, was conducted this study in the Centrale and Kara health regions in Togo, in order not only to assess QOL of PLWHA, according to the WHOQOL-HIV-BREF criteria, but also analyse the factors associated with a good global QOL.

## Main text

### Methods

#### Study design and study population

A cross-sectional study was conducted among HIV-infected patients in two health regions in Togo (Centrale and Kara regions), over a period of 4 months from May to August 2016. Participants to the study were eligible if they were aged 15 years or more, registered in the active file in the selected centers and giving their consent to participate in the study.

#### Setting and sampling

To determine the size of the study population, we used a two-stage sampling procedure taking into account the proportionality criteria related to the number of patients in the active file of PLWHA in the health facilities. In a first stage, we use a random probability sampling of the 30 HIV healthcare facilities in Centrale and Kara regions, ensuring a representation of healthcare centres with a high number of PLWHA. In a minimalist scenario, we made the assumption that a sample of 15% of HIV healthcare centres should be representative of all the healthcare centres in these two regions. So, five HIV healthcare centers were selected for the implementation of this study. At a second stage we carried out a non-probabilistic, convenience sampling, in these five selected HIV healthcare centres, it was proposed to include any PLWHA who visited the healthcare centre for a follow-up from May to August 2016, who met the inclusion criteria and who consented to participate in the study. As before this the prevalence of good quality of life was unknown in that population, it was assumed to be 50% with the precision of 5%. 15% refusal or incomplete data and a design-effect estimated at 2. Based on these hypotheses, we estimated the sample size at 884 HIV-infected patients in the two health regions.

#### Data collection

We used a standardized questionnaire in French to collect data on socio-demographic characteristics, clinical outcomes, ART, sexual activity and on contraception knowledge and its use. The questionnaires were administered during a face to face interview, and were filled by the healthcare workers. Data on HIV status disclosure to the sexual partner were collected only among sexually active PLWHA. To assess QOL, we used the specific instrument developed by WHO, the WHOQOL-HIV BREF [2] which includes 31 items. This instrument, used in other studies in West Africa [8, 11], produces six domain scores: physical, psychological, level of independence, social relationships, environment and spirituality/religion/personal beliefs. All domain scores ranged from 4 to 20. Higher scores in each domain indicated higher quality of life for that domain.

In order to obtain the overall quality of life score, the scores of the six domains are summed and an overall score ranging from 24 (poor overall quality of life) to 120 (better overall quality of life) is obtained.

#### Data’s statistical analysis

In descriptive analysis, mean and standard deviation were calculated for continuous variables and proportions for categorical variables. Our main outcome variable was the global quality of life, which was categorized into a dichotomous variable. We retained 77.3 (which was the mid-range the global score) as a cut off of global score of quality of life. A global score less than or equal to 77.3 was considered as poor global quality of life and a global score higher than 77.3 as good global quality of life. For bivariate analysis, we used Pearson Chi square test or Fisher′s exact test to test significant differences between subgroups. All variables significant in the bivariate analysis at a *p* value < 0.20 were introduced in a multivariate backwards stepwise logistic regression analysis to identify independent risk factors for the dichotomous outcome a good or poor global quality of life. Interactions between the independent variables were tested. All these analyses were performed with 95% confident interval (CI) using SPSS Inc software. Version 17.0 (statistical package for social science, SPSS Inc, Chicago, IL, USA).

### Results

#### Socio-demographic and clinical characteristics

In total, 880 individuals participated in the study, with mean age (SD) of 39.6 (10.1) years. Most of the participants (78.4%) were female, 17.6% had no formal education, 51.7% were living in couple. According to their location, 476 participants (54.1%) were living in Centrale region and 404 (42.1%) in Kara region, while 62.6% of the patients were living in urban area.

Patients were mostly at stage I (51.9%) or at stage II (28.8%) of WHO’s clinical classification. Out of the 880 participants, 796 (90.5%) were on ART among whom 585 (73.7%) had been undergoing ART for 2 years or more. At the last check-up, 71.7% of the participants had a CD4 cells count more than 350 cells/mm^3^ and 431 (49.0) of the participants did not know the partner’s HIV-status.

Most of the patients were followed-up in a public hospital (60.8%), in a health care center with a doctor (86.8%) or in a health care center with a psychologist (59.1%).

#### Health related quality of life

The global score of quality of life was ranged from 42.6 to 112, with a mean (SD) estimated at 86.3 ± (13.3). More than the three-quarters (76.2%) of the participants had a good global quality of life (Table 1).

**Table 1:** Median and mean scores for global quality of life and for six domains (N = 880)

	Physical	Psychological	Level of independence	Social relationships	Environment	Spirituality/religion	Global quality of life
*Statistics*							
Mean (SD)	15.2 (± 3.3)	15.0 (± 3.2)	14.3 (± 2.8)	14.6 (± 2.9)	11.5 (± 1.9)	15.6 (± 3.7)	86.3 (± 13.3)
Median (IQR)	16.0 (13–18)	15.2 (12.8–17.6)	15 (13–16)	15 (13–16)	11.5 (10–13)	16 (13–19)	88.5 (78.2–95.9)
Minimum	5.0	5.6	4.0	6.0	4.0	6.0	42.6
Maximum	20.0	20.0	20.0	20.0	18.0	20.0	112.0

The proportion of patients with a good global quality of life was higher in those with secondary education or more (79.9%; p = 0.035), in those employed in informal sector (83.0%; p < 0.0001) or in those living Kara health region (90.3%; p < 0.0001). In addition, WHO’s clinical stage (p < 0.0001), symptoms’ intensity (p < 0.0001), CD4 cell counts (p = 0.001), ART (p < 0.0001), knowledge of partner’s HIV status (p < 0.0001), type of health care center (p < 0.0001) were also associated with a good global quality of life.

#### Factors associated with a good global QOL

Because of collinearity evidence between being on ART, symptoms’ intensity, CD4 cell counts and the WHO’s clinical stage; only being ART was included in the multivariate analysis. Four factors remained significantly associated with a good global quality of life (Table 2). PLWHA with a secondary education level or more were almost two times more likely to have a good global quality of life (p = 0.018). PLWHA who were living in Kara health region were about four times more likely to have a good global QOL (p < 0.0001). Being on ART multiplied by seven the odds to have a good quality of life. Finally, participants who knew their partner’s HIV sero-status were almost two times more likely to have a good global QOL.

**Table 2:** Participants characteristics and factors associated with good global quality of life (N = 880)

	N (%)	Good global quality of life N = 671 (76.3%)	Logistic regression	
			Univariate OR [95% CI]	Multivariate aOR [95% CI]
*Age*		0.416		
< 25 years	48 (5.5)	35 (72.9)	1	
25–49 years	678 (77.0)	524 (77.3)	1.26 [0.65–2.45]	
50 years and more	154 (17.5)	112 (72.7)	0.99 [0.48–2.05]	
*Gender*		0.718		
Male	190 (21.6)	143 (75.3)	1	
Female	690 (78.4)	528 (76.5)	1.07 [0.74–1.56]	
*Education level*		0.038		
No education	155 (17.6)	109 (70.3)	1	1
Primary	326 (37.0)	243 (74.5)	1.24 [0.81–1.89]	1.14 [0.71–1.82]
Secondary and higher	399 (45.4)	319 (79.9)	1.68 [1.10–2.57]	1.78 [1.10–2.85]
*Profession*		< 0.0001		
Public sector	102 (11.6)	73 (71.6)	1.04 [0.63–1.71]	
Private sector	62 (7.0)	38 (61.3)	0.65 [0.37–1.16]	
Informal sector	435 (49.4)	361 (83.0)	2.01 [1.40–2.88]	
No profession	281 (31.9)	199 (70.8)	1	
*Living in couple*		0.533		
Yes	455 (51.7)	343 (75.4)	1	
No	425 (48.3)	328 (77.2)	1.10 [0.81–1.51]	
*Religion*		< 0.0001		
None	101 (11.5)	78 (77.2)	1	
Islam	288 (32.7)	196 (68.1)	0.63 [0.37–1.06]	
Christianism	491 (55.8)	397 (80.9)	1.25 [0.74 2.09]	
*Residence place*		0.052		
Urban	551 (62.6)	432 (78.4)	1	
Rural	329 (37.4)	239 (72.6)	0.73 [0.53–1.0]	
*Health region*		< 0.0001		
Centrale	476 (54.1)	306 (64.3)	1	1
Kara	404 (45.9)	365 (90.3)	5.2 [3.56–7.6]	4.39 [2.94–6.57]
*WHO’s clinical stage*		< 0.0001		
Stage I	454 (51.9)	382 (84.1)	1	
Stage II	252 (28.8)	161 (63.9)	0.33 [0.23–0.48]	
Stage III and IV	169 (19.3)	123 (72.8)	0.50 [0.33–0.77]	
*Symptoms’ intensity*		< 0.0001		
None	343 (39.0)	306 (89.2)	1	
Moderate	492 (55.9)	348 (70.7)	0.29 [0.20–0.43]	
Severe	45 (5.1)	17 (37.8)	0.07 [0.04–0.15]	
*CD4 cell counts*		0.001		
< 350	249 (28.3)	208 (83.5)	1	
≥ 350	631 (71.7)	463 (73.4)	0.54 [0.37–0.79]	
*ART*		< 0.0001		
Yes	796 (90.5)	647 (81.3)	10.86 [6.55–18.0]	6.99 [4.11–11.9]
No	84 (9.5)	24 (28.6)	1	1
*ART’s scheme*		0.916		
1st line	723 (90.8)	588 (81.3)	1.03 [0.56–1.91]	
2nd line	73 (9.2)	59 (80.8)	1	
*ART duration (years)*		0.993		
< 2	209 (26.3)	170 (81.3)	1	
≥ 2 years	585 (73.7)	476 (81.4)	1.0 [0.67–1.50]	
*Knowledge of partner’s HIV status*		< 0.0001		
No	431 (49.0)	305 (70.8)	1	1
Yes	449 (51.0)	366 (81.5)	1.82 [1.33–2.5]	1.83 [1.28–2.61]
*Type of health care center*		< 0.0001		
Private	345 (39.2)	287 (83.2)	1.95 [1.39–2.73]	
Public	535 (60.8)	384 (71.8)	1	

### Discussion

The aim of this study was to evaluate the QOL through WHOQOL-HIV BREF scale and to identify factors associated with good QOL among HIV-positive patients followed-up in Centrale and Kara health regions.

We found in our study that, HIV-positive patients who were on ART reported better global QOL, whatever the duration of the ART. The effect of the ART on the health of PLWHA and their well-being is well documented in low-income countries [4, 11, 12]. The effect of ART is expressed in PLWHA by the suppression of the HIV replication, and subsequently AIDS-related mortality, morbidity and symptoms are reduced in these patients [12]. PLWHA on ART, for their follow-up, are strongly linked to HIV care clinics, therefore they are likely to receive more counselling, care and treatment from specialized HIV services. These services may impact positively all the domains HRQOL. Similar findings were reported in previous studies. In Burkina Faso, in both in a cross-sectional and longitudinal studies, Bakiono et al. [4, 11] showed that HIV-positive patients on ART were more susceptible to report better score of QOL, even after 1 year of follow-up. In another study conducted in Vietnam, Tran et al. [13] assessed the HRQOL of 1016 HIV-positive patients, using the Vietnamese version of the WHOQOL-HIV BREF and evidenced that patients on ART for more than 1 year were more susceptible to report higher scores of HRQOL.

Indeed, as well documented in several studies, our study revealed that the global quality of life was better in the patients with higher level of formal education. In general, education plays a major role in understanding and communicating information related to well-being. It allows people to have better perception of health, to follow the instructions of health workers in order to maintain good health, thus improving their quality of life. That could also mean that PLWHA with no or lower education may have great difficulty in following the therapeutic and lifestyle recommendations of caregivers. Similar findings were reported in another studies in Uganda [14, 15], in northern Ethiopia [16], in Burkina Faso [4] and in Brazil [17], in which a poor HRQOL was associated mainly with lower education level. However, it is also reported in Lebanon [18] or in Nigeria [3, 19] that individuals with higher level of formal education were likely to have the lowest overall HRQOL score.

The disclosure of the HIV status contribute to improve the global QOL of the participants of this study. HIV disclosure is known to be the first stage of creating a supportive relationship with not only the sexual partner but also with the family members. Thus that could minimize stigmatization and discrimination toward PLWHA facilitate emotional well-being. In previous studies, HIV disclosure or knowing the partner’s HIV status was associated with the HRQOL [19–22]. Our result is consistent with those of a previous study conducted in India by Chandra et al. [22] which demonstrated that the HIV sero-status disclosure was a significant predictor of HRQOL particularly in social and environmental domains of quality of life of the participants.

PWLHA living in Kara health region were more likely to report better QOL than those living in the Centrale. As the HIV’s prevalence reported in the last DHS [23] survey in Togo in 2014 and the national HIV program [24], we observed in our study a great variation in the HIV-related outcomes across the health regions. The HRQOL in PLWHA might be correlated to the HIV-related outcomes, could be considered as a relevant criterion of the quality of the HIV related services in health facilities. The differences across the health regions could be explained by not only socio-cultural factors, but also structural factors. In fact, in Kara health region there are many health facilities well equipped which are able to provide adapted healthcare to PLWHA. In a large multi-national, clinical trial, Safren et al. [25] demonstrated that there were variations of HRQOL of PLWHA across countries and even across heath settings in a country.

### Conclusion

PLWHA interviewed in these two health regions reported a good overall QOL. In addition, the high level of education, ART, living in Kara region and HIV sero-status disclosure was associated with better overall QOL. This QOL could therefore reflect the quality of HIV related care and other interventions that PLWHA benefit in these health facilities. So, it become important to strengthen the caregivers’ competencies on HIV related care, but also implement interventions that could encourage PLWHA to disclose their sero-status.

## Limitations

Some limitations need to be mentioned. First, as the study sample was not representative of the whole country, we refrained to generalize the result of this study across Togo. Second, this study took place in health facilities with little chance of including patients who are less regular in the health centers and may have different characteristics.

## Abbreviations

ART: antiretroviral therapy
QOL: quality of life
PLWHA: people living with HIV/AIDS
WHOQOL-HIV BREF: World Health Organization quality of life scale, brief version
MOS-HIV: medical outcomes surveys HIV
SPSS: statistical package for social sciences
SF-36: Short Form (36) Health Survey
HRQOL: health-related quality of life
CI: confident interval
OR: odds-ratio
aOR: adjusted odds ratio
SD: standard deviation
STI: sexual transmitted infections

## References

[1] ONUSIDA. Fiche d’information—dernières statistiques sur l’état de l’épidémie de sida|ONUSIDA. 2016. http://www.unaids.org/fr/resources/fact-sheet. Accessed 8 June 2017.

[2] O’Connell KA, Skevington SM (2012). An international quality of life instrument to assess wellbeing in adults who are HIV-positive: a short form of the WHOQOL-HIV (31 items). AIDS Behav.

[3] Akinboro AO, Akinyemi SO, Olaitan PB, Raji AA, Popoola AA, Awoyemi OR (2014). Quality of life of Nigerians living with human immunodeficiency virus. Pan Afr Med J.

[4] Bakiono F, Ouédraogo L, Sanou M, Samadoulougou S, Guiguemdé PW, Kirakoya-Samadoulougou F (2014). Quality of life in people living with HIV: a cross-sectional study in Ouagadougou, Burkina Faso. SpringerPlus.

[5] Liping M, Peng X, Haijiang L, Lahong J, Fan L (2015). Quality of life of people living with HIV/AIDS: a cross-sectional study in Zhejiang Province, China. PLoS ONE.

[6] Reis RK, Santos CB, Gir E (2012). Quality of life among Brazilian women living with HIV/AIDS. AIDS Care.

[7] Bello SI, Bello IK (2013). Quality of life of HIV/AIDS patients in a secondary health care facility, Ilorin, Nigeria. Proc Bayl Univ Med Cent.

[8] Osei-Yeboah J, Owiredu WKBA, Norgbe GK, Lokpo SY, Obirikorang C, Alote Allotey E (2017). Quality of life of people living with HIV/AIDS in the Ho Municipality, Ghana: a cross-sectional study. AIDS Res Treat.

[9] Wilson IB, Cleary PD (1995). Linking clinical variables with health-related quality of life. A conceptual model of patient outcomes. JAMA.

[10] Bajunirwe F, Bangsberg DR, Sethi AK (2013). Alcohol use and HIV serostatus of partner predict high-risk sexual behavior among patients receiving antiretroviral therapy in South Western Uganda. BMC Public Health.

[11] Bakiono F, Guiguimdé PWL, Sanou M, Ouédraogo L, Robert A (2015). Quality of life in persons living with HIV in Burkina Faso: a follow-up over 12 months. BMC Public Health.

[12] Joseph Davey D, Abrahams Z, Feinberg M, Prins M, Serrao C, Medeossi B et al. Factors associated with recent unsuppressed viral load in HIV-1-infected patients in care on first-line antiretroviral therapy in South Africa. Int J STD AIDS. 2018. p. 956462417748859. 10.1177/0956462417748859 PMC7053422 29334886

[13] Tran BX (2012). Quality of life outcomes of antiretroviral treatment for HIV/AIDS patients in Vietnam. PLoS ONE.

[14] Stangl AL, Wamai N, Mermin J, Awor AC, Bunnell RE (2007). Trends and predictors of quality of life among HIV-infected adults taking highly active antiretroviral therapy in rural Uganda. AIDS Care.

[15] Mutabazi-Mwesigire D, Katamba A, Martin F, Seeley J, Wu AW (2015). Factors that affect quality of life among people living with HIV attending an urban clinic in Uganda: a cohort study. PLoS ONE.

[16] Tesfay A, Gebremariam A, Gerbaba M, Abrha H (2015). Gender differences in health related quality of life among people living with HIV on highly active antiretroviral therapy in Mekelle Town, Northern Ethiopia. BioMed Res Int.

[17] da Silva J, Bunn K, Bertoni RF, Neves OA, Traebert J (2013). Quality of life of people living with HIV. AIDS Care.

[18] Abboud S, Noureddine S, Huijer HA-S, Deong J, Mokhbat J (2010). Quality of life in people living with HIV/AIDS in Lebanon. AIDS Care.

[19] Bunjoungmanee P, Chunloy K, Tangsathapornpong A, Khawcharoenporn T, Apisarnthanarak A (2014). Quality of life assessment among patients living with HIV/AIDS at a tertiary care hospital in Thailand. Southeast Asian J Trop Med Public Health.

[20] Hasanah CI, Zaliha AR, Mahiran M (2011). Factors influencing the quality of life in patients with HIV in Malaysia. Qual Life Res Int J Qual Life Asp Treat Care Rehabil.

[21] Serovich JM (2000). Helping HIV-positive persons to negotiate the disclosure process to partners, family members, and friends. J Marital Fam Ther.

[22] Chandra PS, Deepthivarma S, Jairam KR, Thomas T (2003). Relationship of psychological morbidity and quality of life to illness-related disclosure among HIV-infected persons. J Psychosom Res.

[23] Ministère de la Planification, du Développement et de l’Aménagement du Territoire (MPDAT), Ministère de la Santé (MS), ICF International. Enquête Démographique et de Santé Au Togo 2013–2014. Rockville: MPDAT, MS et ICF International; 2015. https://dhsprogram.com/pubs/pdf/FR301/FR301.pdf. Accessed 30 Mar 2017.

[24] Programme National de Lutte contre le Sida et les IST (PNLS-IST. RAPPORT ANNUEL 2015 DES ACTIVITES DU PNLS-IST. Lomé: PNLS-IST, 2015:98. http://www.pnls.tg/rapports/RAPPORT%20ANNUEL%20PNLS%202015.pdf. Accessed 26 Aug 2016.

[25] Safren SA, Hendriksen ES, Smeaton L, Celentano DD, Hosseinipour MC, Barnett R (2012). Quality of life among individuals with HIV starting antiretroviral therapy in diverse resource-limited areas of the world. AIDS Behav.

